# Retiform hemangioendothelioma of the breast in a man with ^18^F-flurodeoxyglucose accumulation on positron emission tomography: a case report

**DOI:** 10.1186/s40792-023-01633-8

**Published:** 2023-04-07

**Authors:** Kaoru Ogura, Yoko Shibasaki, Satoshi Honda, Hidetaka Akita, Nobuhiko Aoki, Ja-Mun Chong, Toru Motoi

**Affiliations:** 1 Department of Breast Surgery, Tokyo Metropolitan Toshima Hospital, 33-1 Sakae-Cho, Itabashi-Ku, Tokyo, 173-0015 Japan; 2 Department of Pathology, Tokyo Metropolitan Toshima Hospital, 33-1 Sakae-Cho, Itabashi-Ku, Tokyo, 173-0015 Japan; 3 Department of Radiology, Tokyo Metropolitan Toshima Hospital, 33-1 Sakae-Cho, Itabashi-Ku, Tokyo, 173-0015 Japan; 4 Department of Pathology, Tokyo Metropolitan Cancer and Infectious Diseases Center, Komagome Hospital, 3-18-22 Honkomagome, Bunkyo-ku, Tokyo, 113-8677 Japan

**Keywords:** Male, Breast, Retiform hemangioendothelioma, Positron emission tomography, Magnetic resonance imaging, Angiosarcoma

## Abstract

**Background:**

Retiform hemangioendothelioma (RH) is a rare, intermediate-grade vascular tumor that often arises in the trunk and extremities. The clinical and radiological features of RH remain largely unknown.

**Case presentation:**

A male patient in his 70s presented with shortness of breath on exertion, and computed tomography incidentally revealed a tumor in his right breast. Positron emission tomography (PET) revealed moderate ^18^F-fluorodeoxyglucose (FDG) uptake in the tumor. RH was observed in the resected specimens. Three months after surgery, the patient was free of local recurrence and distant metastasis.

**Conclusions:**

RH was found in the male breast and was accompanied by FDG uptake on PET. PET may be useful in diagnosing RH. Although metastasis is rare in RH, local recurrence may occur, and careful follow-up is required.

## Background

Retiform hemangioendothelioma (RH) is a rare tumor that is locally aggressive and rarely metastasizes to vascular tumors with distinctive hobnail endothelial cell morphology [1]. RH is a disease that affects a wide range of patients, from older adults to the young, is often seen in the extremities and trunk, and occasionally occurs in the penis and scalp [2, 3]. Reports of the radiological features of RH are limited. Incidental detection of RH by positron emission tomography (PET) using ^68^ Ga-DOTATOC has been reported [4]; however, the extent of ^18^F-fluorodeoxyglucose (FDG) accumulation in RH is unknown.

Here, we report the clinical course and pathological features of a case of RH arising in the male breast with ^18^F-FDG accumulation on PET.

## Case presentation

**Fig. 1:**
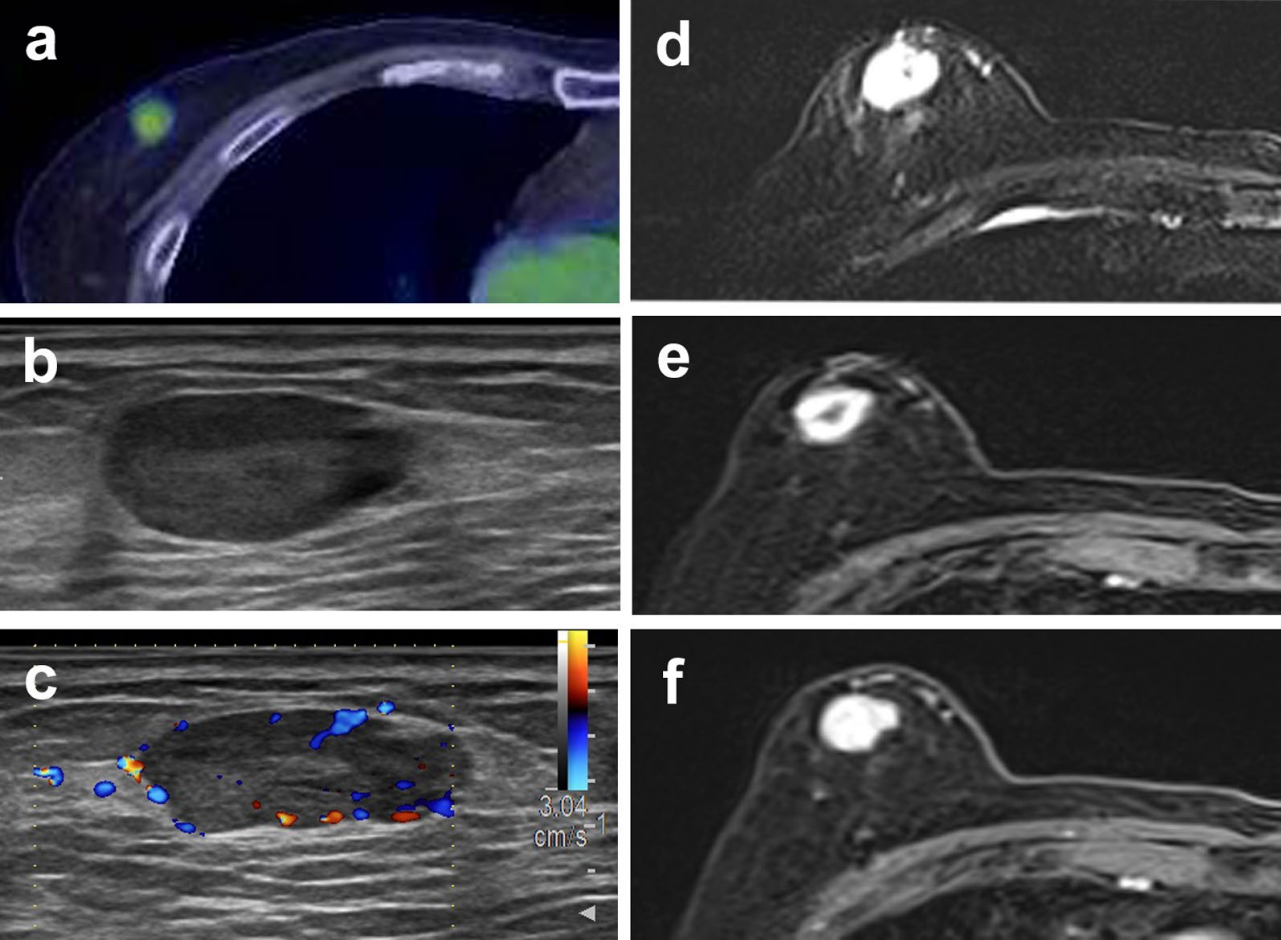
**a** Positron emission tomography showing an accumulation of ^18^F-fluorodeoxyglucose in a mass in the right breast, with a maximum standardized uptake value of 3.11. **b** Breast ultrasonography showed a well-demarcated tumor lesion, a slightly heterogeneous internal echo, and no enhancement of the posterior echo. **c** Color Doppler showed some internal blood flow. **d** T2-weighted images showing high intensity throughout the mass in the upper lateral region of the right breast. **e** T1-weighted gadolinium-enhanced magnetic resonance imaging showing a 2.2-cm-sized smooth-edged mass with early contrast-enhanced margin staining. **f** In the late contrast-enhanced phase, the entire mass is stained and the contrast agent is pooled

A man in his late 70s noticed shortness of breath on exertion 5 months prior. He presented to a previous physician, and computed tomography (CT) revealed moderate emphysema, a nodule in the right lower lobe of the lung, and a mass in the upper lateral part of the right breast. PET showed accumulation of ^18^F-FDG in a nodule in the right lower lobe of the lung and a mass in the right breast with a maximum standardized uptake value of 4.23 and 3.11, respectively (Fig. 1a). The nodule in the right lower lobe was diagnosed as T1bN0M0, Stage IA2 lung cancer. Owing to the presence of respiratory dysfunction and other diseases (bipolar affective disorder), the policy was to follow up without treatment.

The patient had visited our hospital 1 month prior for a detailed examination of the right breast mass. Breast ultrasonography showed a well-demarcated tumor lesion, a slightly heterogeneous internal echo, and no enhancement of the posterior echo. Color Doppler showed some internal blood flow (Fig. 1b, c). T1-weighted gadolinium-enhanced magnetic resonance imaging (MRI) revealed a 2.2 cm smooth-edged mass with an early contrast-enhanced margin in the upper lateral region of the right breast. In the late contrast-enhanced phase, the entire mass was stained and the contrast agent was pooled. T2-weighted images showed a high intensity throughout the mass (Fig. 1d, e, f). These findings suggested vascular tumors, necrosis within the tumor, or vascular malformations as differential diagnoses, in addition to breast cancer. We performed a vacuum-assisted breast biopsy under ultrasound guidance. Histopathological examination of the biopsy specimen showed irregularly dilated and anastomosed reticulated vessels and hobnail-like endothelial cells. In contrast, there was a partial area in which poorly dilated vessels were compactly arranged in alveolar nests. A characteristic reticular pattern and hobnail-like endothelial cells were observed, suggesting an RH. The rate of MIB-1 proliferation was 40% in parts of the alveolar area but < 1% overall. One month after the biopsy, a right partial mastectomy was performed. No standards have been established for the preferred resection margin during surgery with suspected RH. Therefore, the extent of resection of this breast tumor was determined according to surgery for invasive ductal carcinoma of the breast. The resulting histopathology showed no tumor exposure at the resection margin. The final pathological diagnosis of the tumor was RH.

**Fig. 2:**
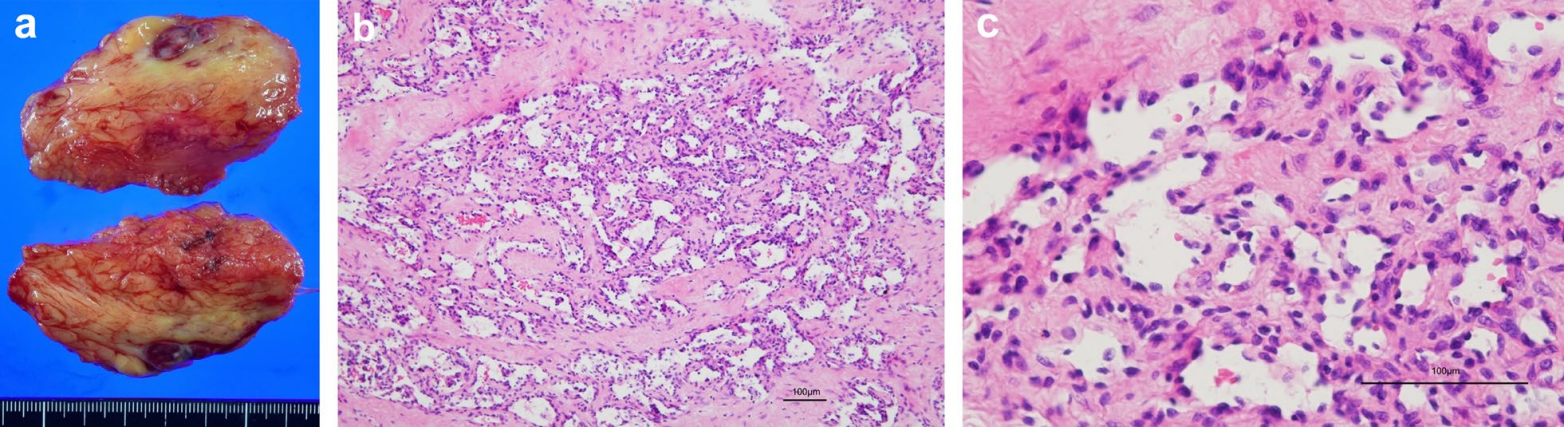
**a** Gross findings: reddish and whitish mosaic nodules with a maximum diameter of 2 cm are observed in the subcutaneous adipose tissue. **b** Histological findings of the resected specimen: abnormal reticular blood vessels with a strong tendency to fuse and proliferate densely. Endothelial cells have small, oval to slightly flattened nuclei, with mildly heterogeneous nuclei and heavily stained chromatin (hematoxylin and eosin, × 400). **c** Hobnail-like protruding image of the lumen and isolated solitary endothelial cells in the vessel lumen (hematoxylin and eosin, × 1000)

A gross examination of the resected specimen revealed multiple reddish tumor nodules measuring 2 cm in size in the subcutaneous adipose tissue (Fig. 2a). Histologically, there was a proliferation of reticular or slit-like abnormal blood vessels, with a strong tendency toward fusion and irregular luminal dilatation with congestion. The tumor endothelial cells had small, oval to slightly flattened nuclei with heavily stained chromatin, and characteristic claw-like projections into the lumen were occasionally observed (Fig. 2b, c). Detached individual endothelial cells were observed in the vascular lumen. Solid growth, lacking an obscure vascular lumen, was also observed. The mitotic figure was obscure. Abnormal vessels were set in a fibrous stroma. A continuous intravascular spread of tumor cells was observed. No neoplastic lesions were observed in the mammary glands.

**Fig. 3:**
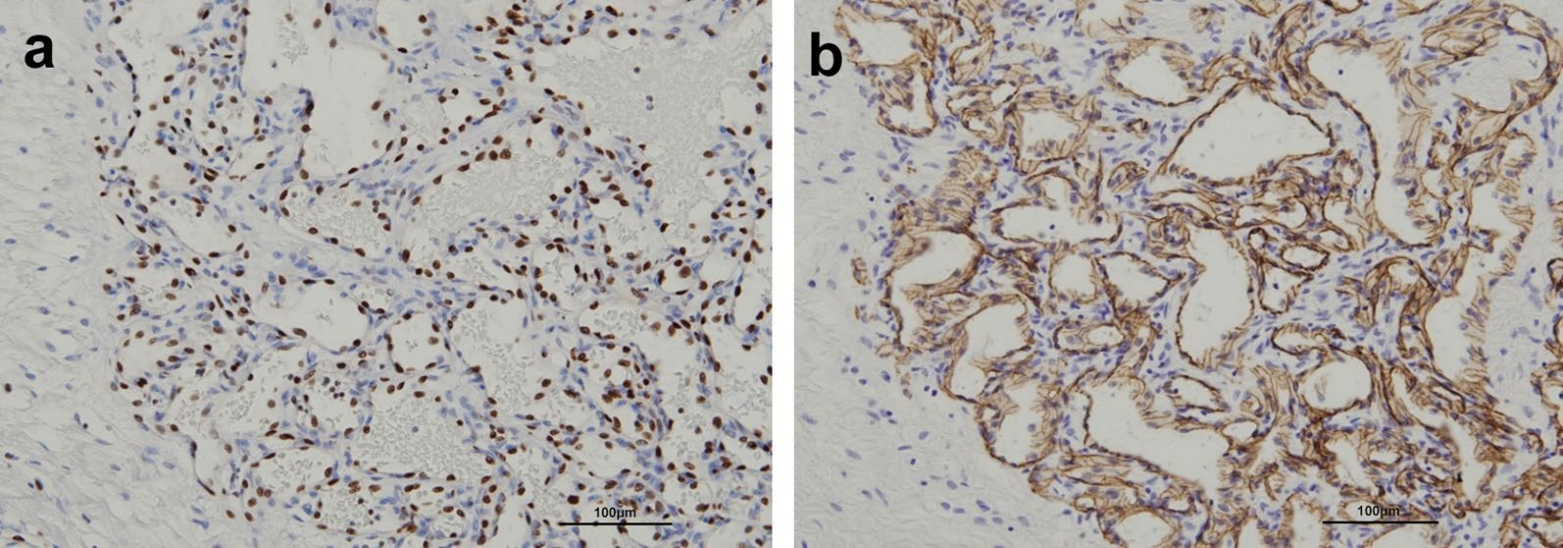
Immunohistochemistry of retiform hemangioendothelioma (**a** ERG and **b** CD31)

Immunohistochemically, the tumor cells were positive for ERG, CD34, factor VIII-related antigen, and CD31, and negative for podoplanin (D2-40) and α-smooth muscle actin, indicating pure vascular endothelial differentiation (Fig. 3). The MIB-1 proliferation rate was ≤ 1%. Pathological diagnosis of RH was made based on characteristic retiform vasculature and claw-like or hobnail tumor cells occupying the entire tumor nodule.

Three months after surgery, the patient was free of local recurrence and distant metastasis.

## Discussion

In this case, RH developed in the male breast, and FDG uptake was observed on PET. RH is a vascular tumor that is considered intermediate-grade and rarely metastasizes. Its epidemiology, prevalent sites, clinical features, and clinicopathological features were first reported by Calonje et al. in 1994 [2]. In this case, the patient showed FDG uptake on PET. This finding is consistent with the high local malignancy of RH. Among vascular tumors, angiosarcoma, which is highly malignant, is important for differential diagnosis and has high FDG uptake on PET. In breast angiosarcoma, high FDG accumulation has been reported in both primary and metastatic lesions [5]. In intermediate-grade vascular tumors, FDG uptake has been reported in pseudomyogenic hemangioendotheliomas [6], and there are multiple reports of FDG uptake in epithelial hemangioendotheliomas [7–10] but not in patients with RH. FDG accumulation on PET may be useful for the diagnosis of RH.

In addition to FDG uptake on PET, MRI revealed staining of the tumor margins in the early phase of T1-weighted contrast enhancement and accumulation of the contrast medium in the late phase. Although these findings are characteristic of vascular tumors, they appeared to be consistent with the imaging findings of RH, an intermediate-grade vascular tumor. Angiosarcoma, a high-grade vascular tumor, has been reported on contrast-enhanced MRI findings in cases involving the breast [11]. The early phase of enhancement stained the majority of the tumor, and the late phase also showed prolonged enhancement. In another report, contrast-enhanced MRI findings of 13 cases of breast angiosarcoma showed heterogeneous enhancement in the early phase and varying degrees of concentric enhancement in the late phase [12]. Although PET and MRI findings may be useful in diagnosing RH, careful resection is necessary considering similar findings in angiosarcoma.

In biopsy specimens, the rate of MIB-1 proliferation was 40% in parts of the alveolar area but < 1% overall. Moreover, it was < 1% in resection specimens. RH has been reported to have low mitotic activity and a low MIB-1 proliferation rate [1, 13, 14]. Our findings indicate that there is heterogeneity in MIB-1 proliferation rates in RH. The diagnosis of a biopsy specimen of a vascular tumor is often challenging, owing to its heterogeneity and overlapping histology. Angiosarcoma is at the center of this issue and may partially contain low-grade histomorphology and retiform vasculature. Composite hemangioendothelioma is another important locally aggressive vascular tumor for differential diagnosis, which often contains areas of RH as well as angiosarcomatous and/or epithelioid hemangioendotheliomatous components. Consequently, a definitive diagnosis of a vascular tumor requires intensive histological examination of the entire tumor in the resected specimens, to avoid overdiagnosis.

RH is a rare tumor, and the fifth edition of the WHO Classification of Tumors of Soft Tissue and Bone (2020) states that only about 40 cases have been reported to date [1]. Although several more case reports have been made since then [15–17], no patient registry has been established, and the frequency of RH remains unknown. The most common site of RH is in the skin or subcutaneous tissues, especially in the lower extremities. In the present case, RH was expressed in the subcutaneous tissue outside the mammary tissue of the male breast. Based on this case and previous reports, RH can occur anywhere in the skin and subcutaneous tissue. As an intermediate-grade tumor, RH is prone to local recurrence, and a case of recurrence 3 months after surgery has been reported [18]. Insufficient information is available regarding time to recurrence and prognosis, and continued follow-up is needed.

In recent years, gene panel testing using next-generation sequencing has revealed the molecular genetic characteristics of rare tumors. Although there have been no reported cases of gene panel testing for RH, *YAP-1* gene rearrangements or *YAP1-MAML2* fusions have been identified in patients with RH and composite hemangioendothelioma [19]. These are observed in young patients, but rearrangements of the *YAP-1* gene have also been reported in RH patients in their 50 s. Genetic examination was not performed in this case, but considering that RH is a rare and life-threatening disease, due to local recurrence, it will be necessary to use a genetic approach to develop treatment methods other than resection in the future.

## Conclusions

We diagnosed a male patient with RH in the breast, accompanied by FDG uptake on PET. It was suggested that PET may help diagnose RH. Since RH is a rare tumor, its clinicopathological and radiological features remain unclear. Further research on this tumor type is required, including genetic aspects.

## Data Availability

The data sets supporting the conclusions of this study are included within the article.

## Abbreviations

RH: Retiform hemangioendothelioma
CT: Computed tomography
PET: Positron emission tomography
FDG: Fluorodeoxyglucose
MRI: Magnetic resonance imaging
